# Impact of Canagliflozin in Patients with Type 2 Diabetes after Hospitalization for Acute Heart Failure: A Cohort Study

**DOI:** 10.3390/jcm10030505

**Published:** 2021-02-01

**Authors:** Ernesto Martín, José López-Aguilera, Rafael González-Manzanares, Manuel Anguita, Guillermo Gutiérrez, Aurora Luque, Nick Paredes, Jesús Oneto, Jorge Perea, Juan Carlos Castillo

**Affiliations:** 1 Department of Cardiology, Hospital Universitario Reina Sofía, 14004 Córdoba, Spain; mircardjla@gmail.com (J.L.-A.); rafaelglezm@gmail.com (R.G.-M.); manuelp.anguita.sspa@juntadeandalucia.es (M.A.); h72gubag@icloud.com (G.G.); auroraluque91@hotmail.com (A.L.); nick_pahu@hotmail.com (N.P.); mjoneto@hotmail.com (J.O.); jorgeponde@hotmail.com (J.P.); juanc.castillo.dominguez.sspa@juntadeandalucia.es (J.C.C.); 2 Maimonides Biomedical Research Institute of Cordoba (IMIBIC), Universidad de Córdoba, 14004 Córdoba, Spain

**Keywords:** heart failure, readmissions, canagliflozin, sodium glucose co-transporter 2 inhibitor, N-terminal pro-B-type natriuretic peptide

## Abstract

Background: Heart failure (HF) is one of the mayor contributors to cardiovascular morbidity and mortality in patients with diabetes. Sodium-glucose cotransporter 2 (SGLT2) inhibitors have demonstrated to reduce the risk of hospitalization for HF in patients with type 2 diabetes mellitus (T2D). We aimed to assess the risk for re-hospitalization in a cohort of patients hospitalized for HF according to whether or not they received canagliflozin at discharge, as well as changes in N-terminal pro–B-type natriuretic peptide (NT-ProBNP) concentration during follow-up. Methods: We conducted a retrospective longitudinal study at a tertiary centre including 102 consecutive T2D patients discharged for acute HF without contraindication for SGLT2 inhibitors. We compared adverse clinical events (HF rehospitalization and cardiovascular death) and NT-ProBNP changes according to canagliflozin prescription at discharge. Results: Among the 102 patients included, 45 patients (44.1%) were prescribed canagliflozin and the remaining 57 (55.9%) were not prescribed any SGLT2 inhibitors (control group). After a median follow-up of 22 months, 45 patients (44.1%) were hospitalized for HF. Most of the rehospitalizations occurred during the first year (37.3%). HF readmission at first year occurred in 10 patients (22.2%) in the canagliflozin group and 29 patients (49.1%) in the control group (hazard ratio (HR): 0.45; 95% confidence interval (CI): 0.21–0.96; *p* < 0.039). A composite outcome of hospitalization for HF or death from cardiovascular causes was lower in the canagliflozin group (37.8%) than in the control group (70.2%) (HR: 0.51; 95% CI: 0.27–0.95; *p* < 0.035). Analysis of NT-ProBNP concentration showed an interaction between canagliflozin therapy and follow-up time (*p* = 0.002). Conclusions: Canagliflozin therapy at discharge was associated with a lower risk of readmission for HF and a reduction in NT-ProBNP concentration in patients with diabetes after hospitalization for HF.

## 1. Introduction

Heart failure (HF) is one of the mayor contributors to cardiovascular morbidity and mortality in patients with diabetes. The incidence of HF in subjects with diabetes is more than double compared with subjects without diabetes [1,2]. Furthermore, various studies showed that the prevalence of diabetes in patients with HF ranges from 25% to 40% and it has also proved as a relevant predictor of prognosis [3,4].

Prior history of hospitalization for HF and the number of hospitalizations during the course of the disease is a strong independent predictor of mortality [5]. Despite dramatic improvements in HF medical therapies and management, readmission rates remain extremely high, with more than 50% of patients readmitted to hospital within 6 months after discharge [6].

Sodium-glucose cotransporter 2 (SGLT2) inhibitors have demonstrated to reduce the risk of hospitalization for HF in patients with type 2 diabetes mellitus in large clinical trials [7,8,9]. Recently, some trials have also shown a potential benefit in patients with stablished HF with reduced ejection fraction (HFrEF) regardless of the presence or absence of type 2 diabetes mellitus (T2D) (DAPA-HF and EMPEROR-REDUCED [10,11]).

The reduction of cardiovascular events does not seem enough in itself to explain this favorable impact in HF. In fact, some glucose-lowering drugs have been associated with a significant reduction of cardiovascular events and no benefit on HF [12,13,14]. Besides, some others antidiabetic agents have been associated with an increased risk of HF [15,16].

The effect of SGLT2 inhibitors in renal protection is one of the mechanisms proposed to explain the reduction of risk of HF. Canagliflozin showed a 30% relative risk reduction in the primary renal composite outcome compared with placebo (CREDENCE trial [17]). In addition to the renal benefit, other theories have been proposed such as improvement in loading conditions, cardiac metabolism and bioenergetics, inhibition of myocardial Na^+^/H^+^ exchange, reduction of cardiac fibrosis or alteration in adipokines and vascular function [12,18,19,20,21,22,23].

The predictors of HF readmissions are well studied and published; socioeconomic status, clinical factors like N-terminal pro-B-type natriuretic peptide (NT-ProBNP) and other biomarkers, nutritional status, diabetes mellitus, renal insufficiency, chronic obstructive pulmonary disease or anemia, systolic blood pressure, New York Heart Association class (NYHA) or defined medical and invasive therapy were independent predictors of readmissions after HF hospitalization [24,25]. However, the readmission and mortality rates of patients with diabetes and hospitalization for acute HF in relation with addition of SGLT2 inhibitors (canagliflozin) to their baseline treatment have not been well studied. The aim of this study was to analyze the benefit of canagliflozin in patients with T2D after hospitalization for acute heart failure.

## 2. Material and Methods

### 2.1. Study Population

This cohort study included all patients with T2D admitted for HF from January 2017 to December 2019 in a single center (University Reina Sofía Hospital, Córdoba, Spain). We excluded patients in whom treatment with SGLT2 inhibitors was contraindicated (estimated glomerular filtration rate (eGFR) ≤ 45 mL/min/1.73 m^2^) and those who had other SGLT2 inhibitors than canagliflozin in their treatment at discharge. HF was defined as the presence of (i) dyspnea at rest or with minimal exertion (NYHA III-IV at admission), (ii) signs of congestion, such as edema, rales, and/or congestion on chest radiograph, (iii) NT-ProBNP ≥ 1400 pg/mL (for patients with atrial fibrillation: NT-proBNP ≥ 2000 pg/mL), and (iv) treated with loop diuretics during hospitalization.

A total of 102 patients were included in the cohort and divided into two groups according to whether canagliflozin was added to their therapy or not: 45 patients in the canagliflozin group and 57 patients in the control group. The addition of canagliflozin and starting dose were left to the criteria of treating physician.

This study was conducted according to the Declaration of Helsinki and was approved by Local Clinical Research Ethics Committee with code GC-15-2017-001. Written informed consent was obtained from all patients.

### 2.2. Data Collection

Clinical and demographic characteristics of all patients were collected during hospitalization. For all patients, we analyzed cardiac biomarkers, renal function (estimated by modification of diet in renal disease (MDRD) formula), glycated hemoglobin, general blood test, electrocardiographic and echocardiographic data. We also analyzed invasive procedures and outcomes during admission. Data of all medical therapy at discharge were collected, with special attention to HF and T2D therapy.

We analyzed adverse events during the first year and last follow-up with review of medical records. Readmissions due to HF and death from cardiovascular causes analysis were performed with a median follow-up time of 22 of months. NT-ProBNP concentrations were collected at 3 months, 6 months and 1 year after hospitalization with laboratory records if available.

### 2.3. Study Objectives

The primary objective of the study was to evaluate the effect of canagliflozin on HF readmissions at 1 year and total follow-up. We secondarily evaluated other clinical predictors of readmission: a composite endpoint of cardiovascular death or HF readmission and changes in NT-ProBNP concentration at 3 months, 6 months and 1 year. Finally, serious adverse events with canagliflozin treatment were assessed.

### 2.4. Statistical Analysis

Continuous variables are expressed as the mean ± SD or median (interquartile range: IQ25–75) and were compared using Student’s t-test or the Mann–Whitney U test, previously using Shapiro–Wilk to test normality.

Categorical variables are presented as counts and percentages and were compared using the chi-square test or Fisher’s exact test, as appropriate.

Hazard ratios (HR) and their 95% confidence interval (CI) for each clinical endpoint were calculated using Cox proportional hazard analysis. The independent effect of canagliflozin and other clinical variables on adverse clinical outcomes was calculated using a Multivariate Cox proportional hazards regression analyses. We included in the multivariate Cox regression model variables with a *p*-value of ≤0.1 in univariate analyses and variables considered clinically relevant were included as covariates. To control for the inherent biases and confounders in the conventional Cox analysis, an inverse probability weighted (IPTW) Cox model was used to estimate the marginal hazard ratio for the primary endpoint.

Survival curves for clinical endpoints and cumulative event rates were generated using Kaplan–Meier estimates.

Changes in NT-ProBNP concentration during follow-up were compared with repeated-measures ANOVA analysis adjusted for age, sex and concentration at discharge.

A value of *p* < 0.05 was considered statistically significant.

Statistical analyses were performed using SPSS, version 20 (IBMCorp., Armonk, NY, USA) and R Statistical Software, version 3.6.2 (R Foundation for Statistical Computing, Vienna, Austria).

## 3. Results

### 3.1. Baseline Characteristics

Table 1 summarizes the baseline clinical characteristics of the patients stratified by canagliflozin treatment at discharge. Of the total of patients who received canagliflozin, 26 (57.8%) were treated with a 100 mg and 19 (42.2%) with a 300 mg dose. At final follow-up we observed that canagliflozin was stopped in 3 (6.7%) patients and 4 (7%) patients were prescribed a different SGLT2 inhibitor in the control group. These patients were included in the analysis. Patients in the canagliflozin group were younger (69 ± 10 vs. 73 ± 11: *p* = 0.04) and there was a lower proportion of women (33.3% vs. 52.6%; *p* = 0.05). However, comorbidities and clinical features of heart failure were well balanced between both groups; 26 (57.8%) patients in the canagliflozin group and 31 (54.4%) patients in the control group had HFrEF, defined as left ventricular ejection fraction (LVEF) ≤ 40%. There were no differences in heart failure treatment at discharge. In terms of glucose-lowering medication, more patients in the control group received dipeptidyl peptidase-4 (DDP4) inhibitors (21.1% vs. 6.7%; *p* = 0.04).

### 3.2. Clinical Outcomes

**Figure 1:**
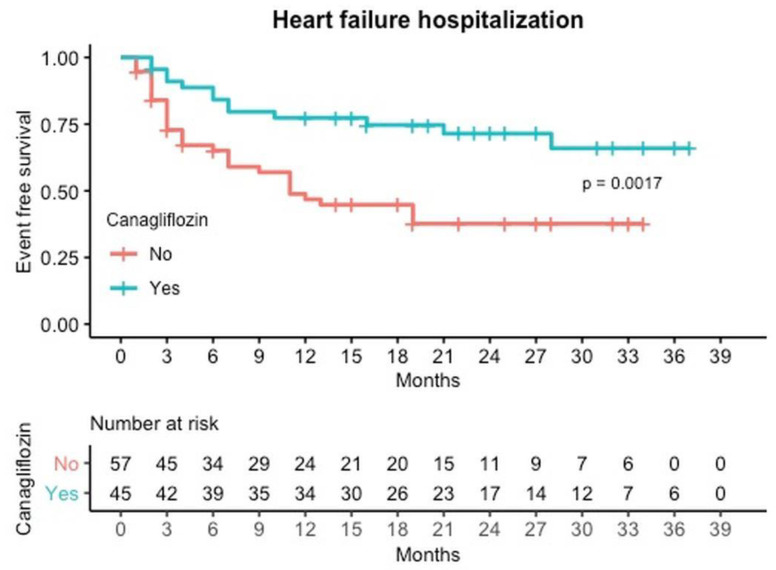
Event free survival curves for Readmission due to heart failure by canagliflozin treatment.

After a median follow-up of 22 months, the primary outcome of hospitalization for HF at first year occurred in 10 patients (22.2%) in the canagliflozin group and 29 patients (49.1%) in the control group (HR: 0.45; 95% CI: 0.21–0.96; *p* < 0.039). Besides, the primary outcome at last follow-up occurred in 13 patients (28.9%) in the canagliflozin group and 32 (56.1%) in the control group (HR: 0.45; 95% CI: 0.23–0.93; *p* < 0.027) (Table 2 or Figure 1), after a median follow-up of 22 months. Most of the readmissions occurred during the first year (37.3%).

**Figure 2:**
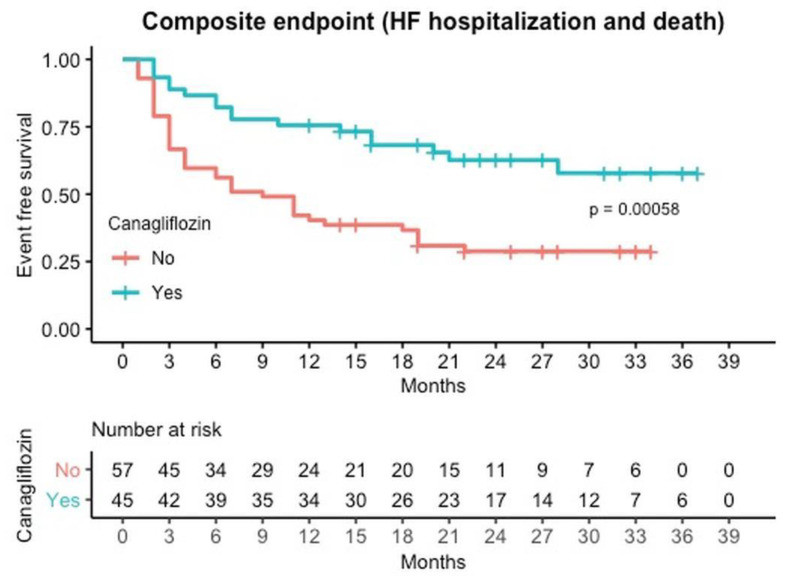
Event free survival curves for composite Mortality or Readmission by canagliflozin treatment.

The secondary composite outcome of hospitalization for HF or death from cardiovascular causes was lower in the canagliflozin group (37.8%) than in the control group (70.2%) (HR: 0.51; 95% CI: 0.27–0.95; *p* < 0.035) (Table 2 or Figure 2). Deaths from cardiovascular causes occurred in 9 patients (20%) in the canagliflozin group and in 23 patients (40.4%) in the control group.

We did not detect serious adverse events among patients who received canagliflozin. Three patients discontinued canagliflozin during follow-up, two of them due to hypotension and one by medical criteria. None of them needed to interrupt the treatment due to worsening renal function. Rate of amputations were similar in both groups, one patient in the canagliflozin group (2.2%) and one patient in the control group (1.8%).

### 3.3. Predictors of Readmissions for Heart Failure

Univariate and multivariate analyses are listed in Table 3. Previous NYHA class (HR: 3.56; 95% CI: 1.55–8.14; *p* < 0.003), previous hospitalization for HF (HR: 3.41; 95% CI: 1.65–7.02; *p* < 0.001) and NT-ProBNP concentration at discharge (HR: 2.8; 95% CI: 1.07–7.36; *p* < 0.036) were independent predictors of readmission at first year; on the contrary, canagliflozin therapy at discharge acted (HR: 0.45; 95% CI: 0.21–0.96; *p* < 0.039) as a protective factor. Coronary artery disease was found to be a significant factor on univariate but not on multivariate analysis.

To account for selection bias and confounders in the conventional Cox analysis, the impact of canagliflozin on HF readmissions was estimated using an IPTW-adjusted Cox model (Table 4). The covariates included in the propensity score model were sex, age, prior HF, coronary artery disease, NYHA class, NT-ProBNP at discharge. By the IPTW-adjusted Cox analysis, the magnitude of effect of canagliflozin remained the same, with HR (95% Cl) of 0.49 (0.25–0.96).

### 3.4. Changes in NT-proBNP Concentration

**Figure 3:**
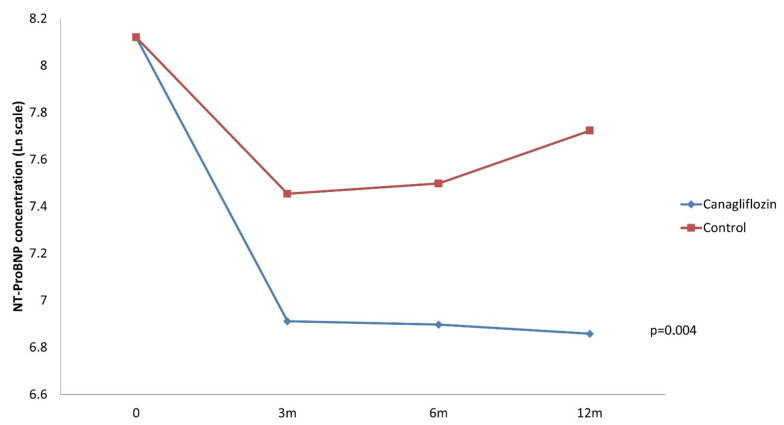
Changes in NT-ProBNP concentration during follow-up. Repeated measures ANOVA analysis adjusted for sex, age and NT-ProBNP concentration at discharge.

Figure 3 summarizes the observed changes in serum NT-ProBNP Napierian logarithmic concentration at discharge, 3 months, 6 months and 1 year. A total of 68 patients (66.7%) had NT-ProBNP concentration available in laboratory records in all periods. Median concentration at discharge in the control group was higher (5036.4 pg/mL) than in the canagliflozin group (3763.5 pg/mL) (*p* = 0.092).

Two-way repeated measures ANOVA adjusted for age, sex and NT-ProBNP concentration at discharge, showed an interaction between canagliflozin therapy and follow-up time (*p* = 0.004). Patients under treatment with canagliflozin experienced greater reductions in NT-ProBNP concentration at 3 months, 6 months and 1 year, compared to concentration at discharge.

## 4. Discussion

The main result of this study was that canagliflozin prescription at hospital discharge for HF in T2D patients was associated with a lower risk of readmission due to HF. These results were consistent regardless of LVEF (≤40% or >40%). Moreover, there was a benefit of canagliflozin with respect to the composite outcome of HF hospitalization and cardiovascular death.

The effect of canagliflozin on readmissions seems to appear since the first months after discharge, as illustrated in the Kaplan–Meir cumulative curves (Figure 1 and Figure 2). This early separation of the survival curves may be explained by the beneficial hemodynamic effects and the improvement in ventricular loading conditions of canagliflozin. SGLT2 and SGLT1 are responsible for over 14% of total renal NaCl reabsorption and are the principal filters of glucose in the proximal tubule. For this reason, inhibition of SGLT2 generates a lasting reduction in the volume of intravascular and extracellular water [26]. Nevertheless, this separation continues through the entire first year, supporting that there might be other mechanisms that account for the sustained favorable effects of canagliflozin [17,18,19,20,21,22].

Pivotal trials with empagliflozin, dapagliflozin and canagliflozin have shown lower rate of admission for HF in patients with T2D [7,8,9]. Dapagliflozin is the first SGLT2 inhibitor that demonstrated a reduction in hospitalization and urgent visit resulting in intravenous therapy for HF in patients with stablished HFrEF [10].

Little data is available about the effect of SGLT2 inhibitors after hospitalization for HF. One pilot randomized study with empagliflozin showed a reduction in a combined endpoint of worsening HF, rehospitalization for HF or death at 60 days [4 (10%) vs. 13 (33%); *p* = 0.014] and proved that it was safe, well tolerated, and with no adverse effects on blood pressure or renal function (EMPA-RESPONSE-AHF [27]). A post-hoc analysis of the EMPA-REG OUTCOME trial with patients who were hospitalized for HF after randomization demonstrated a reduction in readmission rate at 45, 60 and 90 days. The percentages of patients with HF rehospitalization or cardiovascular death within 90 days were 22.1% in the control group and 11.1% in the empagliflozin group [28]. More recently, a cohort study of 104 consecutive patients discharged for acute heart failure and stratified by SGLT-2 inhibitors prescription concluded that these agents were safe, well tolerated, and associated with a reduction in all-cause and cardiovascular deaths [29]. The comparative effectiveness of cardiovascular outcomes in new users of SGLT2 inhibitors (CVD-REAL) 2 study [30], which examined cardiovascular outcomes in a large international cohort with 2,581,980 patients with diabetes and initiated SGLT2 inhibitors versus other glucose-lowering drugs, observed over 441,357 person-years of follow-up, 2646 events of HF hospitalization in the SGLT2 inhibitors group and 3,351 in control group (HR: 0.64; 95% CI: 0.50 to 0.82; *p* = 0.001). Although these published results are similar than those of our study, the rate of readmissions was appreciably higher, 28.9% in canagliflozin group versus 56.1% in control group (HR: 0.45; 95% CI: 0.21–0.96; *p* < 0.039). This fact might be explained by a longer follow-up (median 22 months) and the clinical characteristics of the patients included. To our knowledge, this is the first study that quantified the benefit of canagliflozin after hospitalization for HF.

Potential clinical predictors of HF readmissions are well known. We identified coronary artery disease, NT-ProBNP concentration, previous hospitalization for heart failure and NYHA class as possible risk factors for HF readmission. However, HFrEF was not associated with major rates of readmissions, highlighting the poor clinical outcomes of HF with preserve ejection fraction as well. These results are aligned with those previously reported in literature [31].

We also found that serum concentrations of NT-proBNP, with proven prognostic value for HF and diabetes [24,32], experienced a mayor reduction in the canagliflozin group and those levels were sustained throughout the follow-up period. There are limited data on the effects of canagliflozin on cardiovascular biomarkers. A post-hoc analysis with 666 patients included in the CANVAS Program showed that NT-ProBNP concentrations increased with placebo and minimally with canagliflozin after a 2-year follow-up and from a baseline median of 47 pg/mL. The effect was observed at 26 weeks and persisted over 104 weeks (nominal *p* < 0.05 at weeks 26 and 52, nominal *p* < 0.01 at week 104). In our cohort, baseline NT-ProBNP concentrations were 5036.4 pg/mL in the control group and 3763.5 pg/mL in the canagliflozin group. Consequently, we can compare the real effect in cardiovascular biomarkers of canagliflozin in patients with established HF.

Our beneficial results in terms of rehospitalizations and NT-ProBNP reduction and the lack of serious adverse events are in line with previous research, and suggest that hospitalization for heart failure should be considered as an opportunity for the clinician to initiate canagliflozin treatment in the T2D patient hospitalized for HF at the moment of discharge.

### Limitations

Several limitations of the study should be noted. First, it was not a randomized controlled trial, and it poses the limitations inherent to its retrospective design. The information about the reasons why physicians did not prescribe SGLT2 inhibitors to some patients at discharge was not available. Although the two groups were not totally homogeneous due to slight differences in age and gender proportion, these slight differences may hardly account for the observed results. Moreover, we adjusted for potential confounding factors by multivariable analysis. Other unmeasured, residual variables as well as selection bias could not be completely controlled. The second limitation was the small sample size due to the strict inclusion criteria in this single center study. Nevertheless, the number of events was higher than expected, even though this factor might reduce the strength of our findings.

Thirdly, differences between NT-ProBNP levels at discharge in both groups might influence repeated measures analysis, even after adjustment.

Finally, among patients with HFrEF, use of sacubitril–valsartan was similarly low in both groups, which could be a potential factor for readmissions in this subgroup of patients.

## 5. Conclusions

Canagliflozin therapy at discharge was associated with lower risk of readmission for HF and a reduction in NT-ProBNP concentration in patients with T2D after a hospitalization for HF. Future clinical randomized trials should be performed to confirm our findings.

## Figures and Tables

**Table 1:** Baseline characteristics.

Characteristic	Canagliflozin (*n* = 45)	Control (*n* = 57)	*p*-Value
Age	69 ± 10	73 ± 11	0.04
Female sex	15(33.3%)	30(52.6%)	0.05
Body-mass index (kg/m^2^)	31.9 ± 5.1	30 ± 4.4	0.14
Dyslipidaemia	24(53.3%)	28(49.1%)	0.67
Hypertension	37(82.2%)	48(84.2%)	0.79
Atrial fibrillation or flutter	13(28.9%)	20(35.1%)	0.66
Coronary artery disease	11(24.4%)	17(29.8%)	0.55
Previous stroke	5(8.8%)	0(0%)	0.04
Chronic obstructive pulmonary disease	11(24.4%)	8(14%)	0.18
Previous functional class (NYHA)			
I–II	38(84.4%)	50 (87.7%)	0.7
III–IV	7(15.6%)	7(12.3%)	
Previous hospitalization for heart Failure	15(33.3%)	27(47.4%)	0.15
Clinical features of heart failure			
Ejection fraction (%)	45.4 ± 17.9	49.9 ± 17.8	0.19
Ejection fraction ≤ 40%	26(57.8%)	31(54.4%)	0.73
Ischemic cause	17(37.8%)	16(28.1%)	0.32
Median NT-proBNP (IQR)—pg/ml	3763.5 (1331.4–10,414.6)	5036.4 (2474.2–16,129.1)	0.092
LBBB	7(15.6%)	9(15.8%)	0.86
Killip class on admission			
I–II	35(77.8%)	44(77.2%)	0.94
III–IV	10(22.2%)	13(22.8%)	
Serum creatinine(mg/dl)	1.07 ± 0.3	1.1 ± 0.4	0.92
Estimated GFR (ml/min/1.73 m^2^)	69.7 ± 24.4	68.6 ± 26.3	0.82
Serum potassium (mmol per liter)	4.2 ± 0.5	4.3 ± 0.5	0.47
Hemoglobin (g/dL)	12.7 ± 2	12.3 ± 2.3	0.31
Glycated hemoglobin	7.4 ± 1.5	6.8 ± 2.5	0.16
Device therapy			
Implantable cardioverter-defibrillator	1(2.4%)	4(8.7%)	0.21
Cardiac resynchronization therapy	0(0%)	3(6.5%)	0.1
Heart failure treatment at hospital discharge			
ACEi/ARB inhibitor	38(84.3%)	44(77.7%)	0.35
ARN inhibitor	7(15.6%)	8(14%)	0.83
Beta-blocker	35(78.8%)	45(78.9%)	0.9
MRA	26(57.8%)	30(52.7%)	0.67
Loop diuretic	35(77.7%)	46(80.7%)	0.66
Digoxin	6(13.3%)	14(24.6%)	0.16
Glucose-lowering medication			
Biguanide	35(77.8%)	43(75.4%)	0.78
Sulfonylurea	2(4.4%)	4(7%)	0.58
DPP-4 inhibitor	3(6.7%)	12(21.1%)	0.04
GLP-1 receptor agonist	1(2.2%)	5(8.8%)	0.16
Insulin	12(26.7%)	22(38.6%)	0.26

Numeric values are expressed as median (Interquartile range) or number (percentage %). IQR: interquartile range. ACE denotes angiotensin-converting enzyme, ARB angiotensin receptor blocker, ARN angiotensin receptor neprilysin, MRA mineralocorticoid receptor antagonist, DPP-4 dipeptidyl peptidase 4, GFR glomerular filtration rate, GLP-1 glucagon-like peptide 1, LBBB left bundle branch block, MRA mineralocorticoid receptor antagonist, NT-proBNP N-terminal pro-B-type natriuretic peptide, and NYHA New York Heart Association.

**Table 2:** Adverse events according to Canagliflozin treatment.

Adverse Event	Canagliflozin	Control	Ratio or Difference	*p*-Value
	(*n* = 45)	(*n* = 57)	(95% CI)	
Readmission during first year	10(22.2%)	29(49.1%)	HR 0.45(0.21–0.96)	0.039
Readmission during follow-up	13(28.9%)	32(56.1%)	HR 0.45 (0.23–0.91)	0.027
Number of readmissions at follow-up	0.5 ± 0.9	1.3 ±1.8	Dif 0.8 (0.3–1.4)	0.009
All-mortality or readmission	17(37.8%)	40(70.2%)	HR 0.51 (0.27–0.95)	0.035
Change in Glycated hemoglobin at 1yr. (%)	−0.06 ± 1.8	0.06 ± 2.1	Dif −0.12 (−1.06–0.83)	0.81

Hazard ratios (HR) and their 95% confidence interval (CI) for each clinical endpoint was calculated using a Multivariate Cox proportional hazards regression analyses adjusted for age, sex, NYHA class, previous hospitalization for heart failure, coronary artery disease and Log NT-ProBNP at discharge.

**Table 3:** Univariate and multivariate cox regression analysis for risk factors for readmission due to heart failure at first year.

	Univariate		Multivariate	
	HR(95% CI)	*p*-Value	HR(95% CI)	*p*-Value
Age	1.02(0.99–1.05)	0.31		
Gender (male)	1.21(0.63–2.32)	0.57		
Body-mass index (kg/m^2^)	1.03(0.94–1.13)	0.5		
NYHA (I–II vs III–IV)	2.23(1.01–4.91)	0.046	3.56(1.55–8.14)	0.003
Previous hospitalization for heart Failure	3.0(1.55–5.82)	0.001	3.41(1.65–7.02)	0.001
Coronary artery disease	1.75(0.91–3.35)	0.093	1.24(0.58–2.69)	0.58
Ejection fraction ≤ 40%	1.11(0.58–2.11)	0.75		
Atrial fibrillation	1.08(0.54–2.18)	0.82		
Chronic obstructive pulmonary disease	1.69(0.80–3.57)	0.17		
Systolic blood pressure (mmHg)	1(0.99–1.01)	0.75		
Log NT-proBNP (pg/mL)	3.26(1.35–7,87)	0.009	2.8(1.07–7.36)	0.036
Canagliflozin treatment	0.35(0.17–0.73)	0.005	0.45(0.21–0.96)	0.039

**Table 4:** Impact of canagliflozin on readmission for HF at first year by conventional and IPTW-adjusted Cox regression analysis.

	HR	*p*-Value
Models	(95% CI)	
Univariate	0.35(0.17–0.73)	0.005
Multivariate	0.45(0.21–0.96)	0.039
IPTW-adjusted	0.49(0.25–0.96)	0.038

Hazard ratios (HR) and their 95% confidence interval (CI) for HF readmission at first year calculated using an inverse probability of treatment weighting (IPTW)- adjusted cox proportional hazards regression analyses adjusted for age, sex, NYHA class, previous hospitalization for heart failure, coronary artery disease and Log NT-ProBNP at discharge.

## Data Availability

The data presented in this study are available on request from the corresponding author. The data are not publicly available due to ethical reason.
